# Sensitivity to cdk1-inhibition is modulated by p53 status in preclinical models of embryonal tumors

**DOI:** 10.18632/oncotarget.3908

**Published:** 2015-05-11

**Authors:** Melanie Schwermer, Sangkyun Lee, Johannes Köster, Tom van Maerken, Harald Stephan, Angelika Eggert, Katharina Morik, Johannes H. Schulte, Alexander Schramm

**Affiliations:** ^1^ Department of Pediatric Oncology and Hematology, University Children's Hospital Essen, Essen, Germany; ^2^ Department of Computer Sciences, TU Dortmund University, Dortmund, Germany; ^3^ Department of Genome Informatics, University Hospital Essen, Essen, Germany; ^4^ Centre for Medical Genetics, Ghent University Hospital, Ghent, Belgium; ^5^ Charite University Medicine, Berlin, Germany; ^6^ Centre for Medical Biotechnology, University Duisburg-Essen, Essen, Germany; ^7^ Translational Neuro-Oncology, West German Cancer Center, University Hospital Essen, University Duisburg-Essen, Essen, Germany; ^8^ German Cancer Consortium (DKTK), Heidelberg, Germany; ^9^ German Cancer Research Center (DKFZ), Heidelberg, Germany

**Keywords:** cell cycle, cyclin-dependent kinases, cdk1/CCNB1 complex

## Abstract

Dysregulation of the cell cycle and cyclin-dependent kinases (cdks) is a hallmark of cancer cells. Intervention with cdk function is currently evaluated as a therapeutic option in many cancer types including neuroblastoma (NB), a common solid tumor of childhood. Re-analyses of mRNA profiling data from primary NB revealed that high level mRNA expression of both cdk1 and its corresponding cyclin, CCNB1, were significantly associated with worse patient outcome independent of MYCN amplification, a strong indicator of adverse NB prognosis. Cdk1 as well as CCNB1 expression were readily detectable in all embryonal tumor cell lines investigated. Pharmacological inhibition or siRNA-mediated knockdown of cdk1/CCNB1 induced proliferation arrest independent of MYCN status in NB cells. Sensitivity to cdk1 inhibition was modulated by TP53, which was demonstrated using isogenic cells with wild-type TP53 expressing either dominant-negative p53 or a short hairpin RNA directed against TP53. Apoptosis induced by cdk1 inhibition was dependent on caspase activation and was concomitant with upregulation of transcriptional targets of TP53. Our results confirm an essential role for the cdk1/CCNB1 complex in tumor cell survival. As relapsing embryonal tumors often present with p53 pathway alterations, these findings have potential implications for therapy approaches targeting cdks.

## INTRODUCTION

Targeting the machinery that regulates the aberrant cell cycle associated with malignant tumor growth is currently regarded as a promising anti-cancer strategy. Genetic alterations in cell cycle control genes have frequently been identified in childhood tumors first, with Rb1 as one of the most prominent examples [1, 2]. Among embryonal tumors, neuroblastoma (NB) is a prototypic malignancy with marked heterogeneity of clinical courses ranging from very aggressive behavior and poor patient outcome to spontaneous regression even without treatment [3]. Intensity of NB therapy depends on clinical characteristics such as age at diagnosis and metastatic spread of the disease, but also on molecular factors, most prominently the amplification status of the MYCN oncogene (reviewed in [4]). MYCN amplification/mutation is the most frequent genetic aberration in NB affecting a single gene, which has been confirmed by genome-wide mutational analyses [5]. Enhanced MYCN gene dosage is associated with early disease relapse and unfavorable prognosis. Several lines of evidence have also pointed to an intricate link of MYCN with proteins of the cell cycle machinery, resulting in synthetic lethality of CDK inhibition in MYC(N)-dependent cancers [6, 7]. It thus was hypothesized that interfering with MYCN functions will offer a route to tackle the most aggressive forms on NB and other MYCN-dependent cancers. As MYCN is a transcription factor with a wide range of target proteins and functions, it has been enigmatic for decades, which strategy would be optimal to interfere with MYC(N) functions. Recently, inhibition of Brd proteins, most notably Brd4, has been shown to be effectively down-regulating MYCN functions at least in preclinical models of embryonal tumors including medulloblastoma and neuroblastoma [8, 9]. Still, the clinical usefulness of these strategies has yet to be proven.

Here, we re-analyzed mRNA array profiling data obtained for > 100 primary NB to search for cell-cycle regulated genes correlating with patient outcome and MYCN status. Among these candidate genes, we identified the cell cycle regulators Cyclin B1 (CCNB1) and the cell cycle dependent kinase 1 (cdk1) as intricately linked to patient's prognosis. It is well known that cdk1 is overexpressed in a variety of tumor entities including prostate cancer and oral squamous cell carcinoma [10, 11]. Mechanistically, high levels of cdk1 promote tumor growth by stabilizing HIF1α [12] and contribute to neoplastic transformation by phosphorylation of YAP [13]. Selective inhibition of cdk1 function by small molecules such as RO-3306 is feasible [14] and cdk1 inhibition had synergistic effects in combination with PARP inhibition in preclinical models of breast cancer [15]. In the study presented here, we investigated the effects of CCNB1/cdk1 knock down by siRNA and analyzed the consequences of RO-3306 mediated cdk1 inhibition as a function of variable expression of MYCN and p53 in preclinical models of embryonal tumors including neuroblastoma, medulloblastoma and rhabdomyosarcoma.

## MATERIALS AND METHODS

### Expression profiling of primary human NB

Affymetrix HuEx1.0 exon array data (GEO acc. No GSE32664) were re-analyzed for the purpose this study. The 101 NB samples used were from tumor banks in Essen and Ghent [16]. Treatment protocols and patient selection have been previously described [17]. In brief, we ensured a representative distribution of tumor stages (stage 1: *n* = 23; stage 2: *n* = 7; stage 3: *n* = 11; stage 4: *n* = 42; stage 4s: *n* = 18), with 75% of the patients (*n* = 76) older than one year at the time of diagnosis. The mean age at diagnosis was 607 days and MYCN amplification occurred in 19 patients. Microarray data were analyzed using the web-based frontend R2 (r2.amc.nl).

### Cell lines

Human neuroblastoma cell lines with high MYCN levels (IMR32, NGP, NLF, WAC2) or low MYCN levels (NB69, SHEP, SK-N-FI) were used. RH-41 was established from a xenografted lung metastasis of alveolar rhabodomyosarcoma [18]. WAC2 is a subclone of SHEP cells engineered for stable overexpression of MYCN [19]. Inducible MYCN activation was achieved using SHEP MYCN-ER cells. Briefly, nuclear translocation and activation of MYCN in SHEP MYCN-ER cells expressing a fusion protein of MYCN and the estrogen-responsive domain of the estrogen receptor was induced by addition of 200 nM 4-OHT for indicated time points as described [20]. Down-regulation of p53 in wt-TP53 NB cell lines IMR32 and NGP was facilitated by an shRNA directed against human p53, while a shRNA directed against murine p53 served as negative control [21]. HD-MB3 medulloblastoma cells expressing a dominant-negative variant of p53 (HD-MB3 p53-dn) cells were generated by transfecting HD-MB3 with pMSCV-puro-p53DD, and selected for stable transfectants with 2 μg puromycin/ml medium [8]. MYCN was down-regulated in a MYCN-amplified cell line, IMR5, by using a tet-inducible two vector system [22]. In these cells, designated IMR5-shMYCN, addition of tetracycline (1 μg/ml) to the culture media induced ectopic overexpression of an shRNA directed against NMYC [22]. All cell lines were cultivated in RPMI 1640 containing 10% FCS and antibiotics as previously described [23]. Identity of tumor cell lines was confirmed by STR genotyping. The human fibroblast cell line NHDF served as non-tumorigenic control.

### Gene knockdown using small interfering RNAs (siRNAs)

Cells were transfected with 50 nM siRNA directed against either CCNB1 or cdk1 (Qiagen, Hilden, Germany) using HiPerFect transfection reagent (Qiagen). As a control, the cells were transfected with a non-targeting siRNA (D-001210-01-05, Thermo Scientific Dharmacon, Waltham, MA). Down-regulation of target mRNA was validated by semi-quantitative real-time PCR.

### Cell cycle analysis

Cells were cultivated in the presence of the cdk1-inhibitor, RO-3306, for 24 h or 48 h, harvested, and stained with propidium iodide as described in [24]. The DNA content as a function of the cell cycle phase was analyzed using a FC500 Flow Cytometer (Beckman Coulter).

### Cell viability assays

Cells were seeded in triplicates into 96 well plates to adhere. After 24 hours the cells were treated with either a cdk inhibitor, RO-3306, or siRNA for 48 h. Cell viability was determined by a MTT (3-(4, 5-dimethylthiazol-2-yl)-2, 5-diphenyltetrazoliumbromide) assay.

### Western blot

Cells were washed with cold PBS and lysed in RIPA buffer containing proteases and phosphatase inhibitors (Roche, Penzberg, Germany). Gel electrophoresis, transfer to nitrocellulose membranes, blotting and visualization was performed as described [25]. The membranes were probed with the following antibodies and dilutions: p53 (1:500; Santa Cruz), p21 (1:1000; Cell Signaling), CCNB1 (1:500; Abnova), cdk-1 (1:500; Milipore), NMYC (1:500: Cell Signaling), pRb-Ser807/811 (1:2000; Cell Signaling), PP1α and pPP1α-Thr320 (1:500; Cell Signaling).

### Real-time PCR and semiquantitative PCR

RNA was isolated from cells using the High Pure RNA isolation Kit (Roche). The cDNA was synthesized with the Transcription First Strand cDNA Synthesis Kit (Roche). For semiquantitative PCRs 100 ng cDNA was used and GAPDH was co-amplified as a control. Real-time PCRs was performed using predesigned primers (Qiagen) and monitored using SYBR green fluorescence on a StepOnePlus Real-Time PCR system (Life Technologies). Target gene expression was calculated using the delta Ct method using GAPDH as internal control.

### Apoptosis and caspase assays

Apoptosis was monitored following cdk inhibition for 48 h using the Cell Death Kit Plus (Roche) allowing for the specific determination of mono- and oligonucleosomes as consequence of DNA fragmentation. Caspase activity was determined using luminogenic Caspase 8 or Caspase 9 substrates (Caspase Glo Assay, Promega), respectively, according to the manufacturer's instructions. For rescue experiments, cells were seeded on 12 well plates for 24 h and then treated with either RO-3306 in the presence or absence of the pan-caspase inhibitor, Q-VD-OPh (Calbiochem), for 48 h.

### Statistical analyses

Statistical significance of differences between treatment groups was determined using a Student *t*-test implemented in GradhPad Prism^®^ 5.0 (GraphPad Software, San Diego, CA), which was also used for graphical representation and calculation of standard deviation.

## RESULTS

### Cdk1 and CCNB1 mRNA expression are correlated with poor outcome independent of MYCN amplification in neuroblastoma

**Figure 1:**
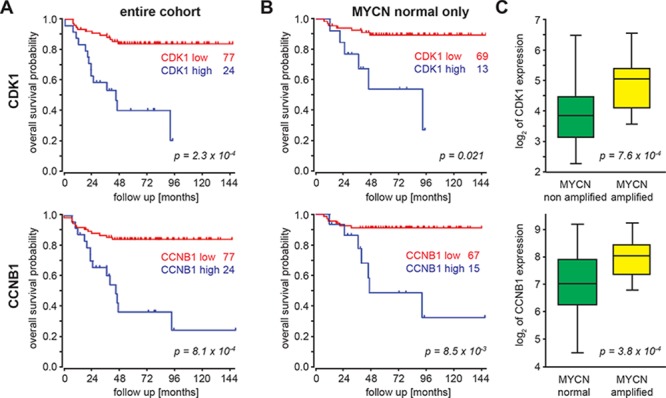
CCNB1 and cdk1 mRNA expression correlate with a poor outcome and MYCN expression in neuroblastoma **A, B.** Kaplan Meyer analyses of 101 primary NB revealed a significant correlation between overall survival and both cdk1 and CCNB1 mRNA expression. The cohort was split into two groups with either “high” or “low” cdk1/CCNB1 expression and the numbers depict the respective group size, when the entire cohort (A) or the subset with normal MYCN status (B) were analyzed. P-values were adjusted for multiple testing of different group sizes using a Bonferroni correction implemented in the R2 visualisation module (accessible at http://r2.amc.nl). **C.** Tumors harboring a MYCN amplification have significantly higher cdk1/CCNB1 expression compared to MYCN non-amplified tumors.

Data obtained from mRNA profiling of 101 primary neuroblastoma were re-analyzed for correlation of cell cycle genes with outcome and the MYCN amplification status. High expression of cdk1 or CCNB1 was linked to significantly worse outcome in the entire cohort (Figure 1A), and this was independent of the MYCN status (Figure 1B). Nevertheless, both cdk1 expression and CCNB1 expression were significantly higher in MYCN-amplified tumor (Figure 1C). Additionally, survival probabilities were significantly lower in tumor with high expression of cdk2 (*p* = 0.006), cdk4 (*p* = 0.003) or cdk6 (*p* = 0.045, Supplementary Figure S1A). Of these genes, only cdk4 was significantly associated with MYCN amplification status (*p* = 0.0002, Supplementary Figure S1B). As previously reported, upregulation of cdks is common in aggressive neuroblastoma [7, 26], but only CCNB1/cdk1 were predictive of outcome both in the entire cohort as well as in the MYCN normal group when the Cyclin/cdk pairs were considered.

### Cdk1 and CCNB1 are highly expressed in neuroblastoma cell lines

**Figure 2:**
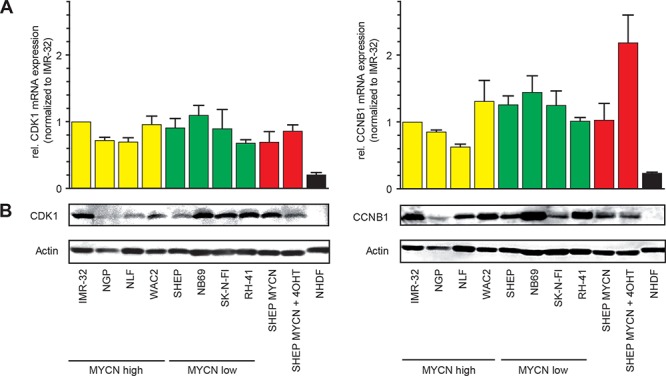
Cdk1 and CCNB1 are highly expressed in neuroblastoma cell lines **A.** CCNB1 and cdk1 mRNA-levels were analyzed by semi-quantitative PCR in seven neuroblastoma cell lines (IMR32, NGP, NLF, WAC2, SHEP, NB69, SK-N-FI) and in the rhabdomyosarcoma cell line RH-41. Expression was normalized to IMR32 cells and compared to the human fibroblast cell line (NHDF). Cell lines were grouped according to their MYCN expression status (yellow = MYCN high, green = MYCN low). In SHEP-MYCN cells, MYCN can be targeted to the nucleus and thus activated by addition of 4-OHT (marked in red). **B.** Western Blot analyses revealed higher protein levels of cdk1 and CCNB1 in the tumor cell lines compared to NHDF cells. MYCN activation by 4-OHT in SHEP-MYCN cells did not result in elevated CCNB1 or cdk1 expression, although CCNB1 mRNA expression was higher compared to controls.

Expression of both cell cycle regulators, cdk1 and CCNB1, were analyzed on mRNA and protein level in a panel of NB cell lines presenting either with low MYCN levels (NB69, SK-N-FI, SHEP) or with MYCN amplification/overexpression (IMR-32, NGP, NLF, WAC2). Both mRNA and protein levels of cdk1 and CCNB1 were elevated in all NB cell lines and the human rhabdomyosarcoma cell line, RH-41, when compared to a human fibroblast cell line (NHDF, Figure 2). No significant correlation between the MYCN status and Cdk1/CCNB1 expression could be detected (Supplementary Figure S2). Of note, in SHEP cells engineered to activate MYCN upon addition of 4-OHT (SHEP MYCN-ER), CCNB1 mRNA (Figure 2A) but not protein levels (Figure 2B) were elevated compared to controls. Induction of shRNA-mediated down-regulation of MYCN in a MYCN-amplified cell line, IMR5, resulted in concomitant down-regulation of both Cdk1 and CCNB1 (Supplementary Figure S3). Thus, MYCN is a sufficient but not necessary regulator of CCNB1/cdk1 expression, and upregulaton of CCNB1 and cdk1 is a common feature of neuroblastoma cell lines irrespective of their MYCN status.

### Knock-down of CCNB1 or cdk1 by specific siRNAs causes a decrease in NB cell proliferation

**Figure 3:**
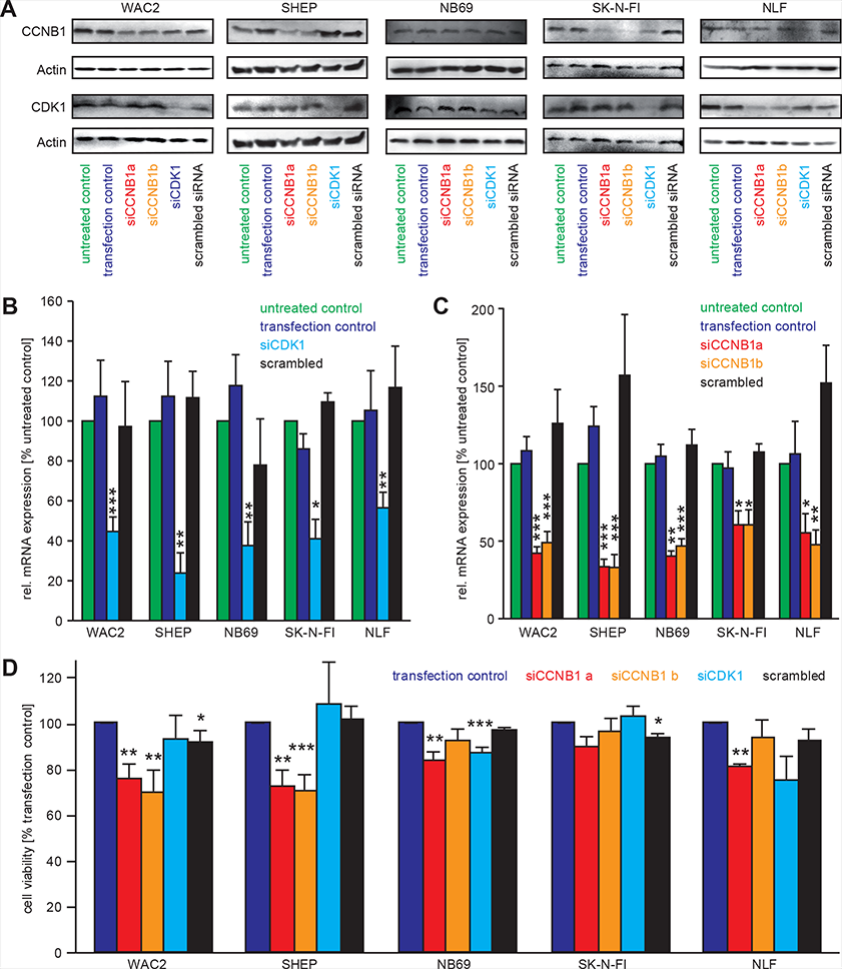
siRNA mediated knock-down of cdk1 and CCNB1 causes a reduction of cell viability **A–C.** Transfection with siRNA against cdk1 (siCDK1) and CCNB1 (siCCNB1a, siCCNB1b) specifically downregulated the protein evels of the respective targets as revealed by Western Blot analyses. In (B) and (C), semi-quantitative real-time PCR for CDK1 and CCNB1 confirmed that down-regulation of the respective mRNAs was also specific when compared to the controls. Values are presented as normalised to untreated control (“untr. ctrl.”). “transfection control” contained the transfection reagents only, while in “scrambled siRNA” an unrelated siRNA was used. **D.** Cell viabilty was measured by MTT assays after 48 h following siRNA transfection. Significant differences between transfection control and knockdown cells are shown in the diagram as follows ***: *p* < 0.001; **: *p* = 0.001–0.01; *: *p* = 0.01−0.05

Transient downregulation of CCNB1 or cdk1 was achieved by sequence-specific siRNAs in a panel of five neuroblastoma cell lines. Both mRNA and protein levels of CCNB1 and cdk1 were significantly and specifically decreased 48 h after transfection (Figure 3A–3C). Cell viability after knock-down of CCNB1 was significantly reduced in SHEP and WAC2 cells, while reduction of cell viability upon knock-down of cdk1 (Figure 3D) was only significant in NB69 cells. A trend for reduced viability was present in all other cell lines, with the exception of the p53-mutated cell line, SK-N-FI, in which cdk1 or CCNB1 downregulation had no effect (Figure 3D).

### The cdk1 inhibitor, RO-3306, causes a significant reduction of cell viability in neuroblastoma cell lines

**Figure 4:**
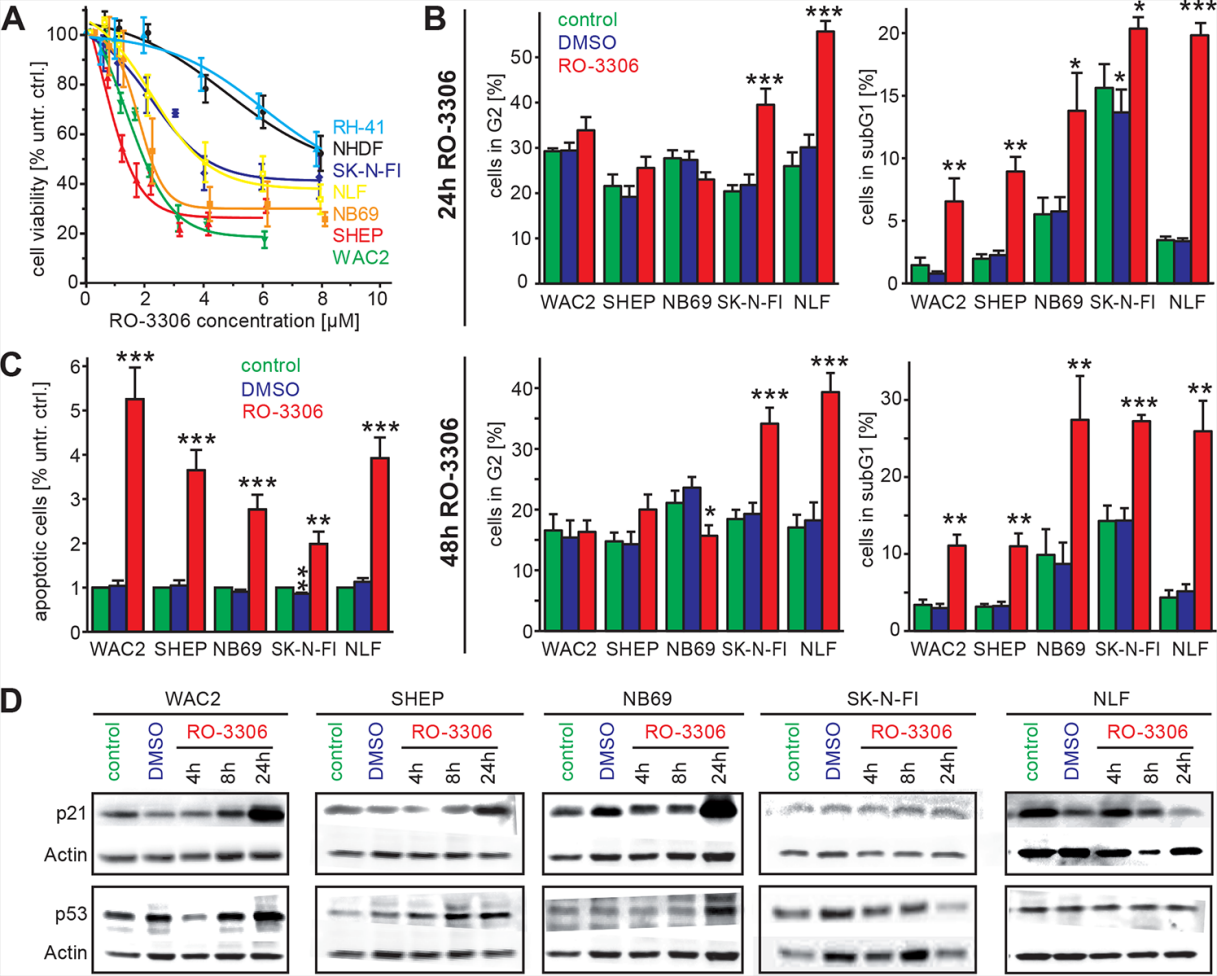
Inhibition of cdk1 by a small molecule inhibitor, RO-3306, induces apoptosis and causes an activation of the p53 signaling pathway **A.** Inhibition of cdk1 by RO-3306 caused decreased cell viability after 48 h in all NB cell lines investigated and also in the rhabdomyosarcoma cell line RH-41 in a concentration dependent manner. The human fibroblast cell line, NHDF, and the cell lines harboring p53 mutation (SK-N-FI, RH-41) were more resistant to RO-3306 than the tumor cell lines with p53 wild type. **B.** Cell cycle distribution was analyzed 24 h and 48 h after RO-3306 treatment. Only cells harboring a p53 mutation (SK-N-FI, NLF) presented with significantly higher fraction of G2 phase (left side), while all cell lines had significantly elevated subG1 indicative of apoptosis. **C.** Significant induction of apoptosis by RO-3306 could be confirmed in all cell lines by a colorimetric assay detecting nucleosomes. **D.** Activation of the p53 pathway in p53 wt cells shown by increased p53 and p21 protein levels. Significant differences between untreated cells and inhibitor treated cells are depicted in the entire diagram using the following code “***”: *p* < 0.001; “**”: *p* = 0.001−0.01; “*”: *p* = 0.01−0.05.

To further characterize the impact of the cdk1/CCNB1 axis on NB cell survival, we used a previously described cdk1 inhibitor, RO-3306 [14]. The effect of RO-3306 on cell viability was analyzed in the same cell line panel as described above. Additionally, a human fibroblast cell line (NHDF) as well as a rhabdomyosarcoma cell line, RH-41, were included for evaluation of tumor cell specific effects. All cell lines responded to RO-3306 treatment by decreased cell viability in a concentration dependent manner (Figure 4A, Table 1). Interestingly, both the rhabdomyosarcoma cells as well as the non-tumorigenic NHDF cells were more resistant to RO-3306 than the NB cells in our panel. IC50-values in NB cells ranged between 1.3 μM and 4 μM, while RH-41 cells had an IC50-value > 8 μM. In isogenic NB cells with low (SHEP) or high levels of MYCN (WAC2) response to RO-3306 was comparable (IC50_48 h_:1.3 μM and 1.8 μM, respectively).

**Table 1:** Molecular characteristics of cell lines used in this study Displayed are the p53 status, the MYCN status of cells and subclones and their sensitivity to RO-3306, a cdk1-inhibitor and to a pan cdk inhibitor, JNJ-7706621, used throughout this study. In the column “p53 status” “knock-down” depicts cell lines with expression of a shRNA directed against p53, while “dominant neg. form” refer to HDMB-3 cells expressing a dominant negative variant of p53. In the column “MYCN status”, cells are divided into the categories “amplified” or “non-amplified” with the exception of WAC2, which is a stably MYCN-overexpressing subclone of SHEP.

Cell line	IC50 RO3306	IC50 JNJ-7706621	p53 status	MYCN status
WAC2	1.80 μM	0,6 μM	wt	high
SHEP	1.30 μM	1 μM	wt	single-copy
NB69	2.30 μM	2,3 μM	wt	single-copy
SK-N-FI	4.00 μM	4,1 μM	mut.	single-copy
NLF	3.80 μM	3,4 μM	mut.	ampl.
RH-41	> 8.0 μM	2,8 μM	mut.	single-copy
IMR-32	1.6 μM	1,3 μM	wt	ampl.
IMR-32 LV-m-p53	1.6 μM	1,3 μM	wt	ampl.
IMR-32 LV-h-p53	2.4 μM	2,1 μM	knock-down	ampl.
NGP	4.0 μM	3,1 μM	wt	ampl.
NGP LV-m-p53	4.0 μM	3,1 μM	wt	ampl.
NGP LV-h-p53	6.0 μM	3,1 μM	knock-down	ampl.
HDMB-3	1.8 μM	1,6 μM	wt	ampl.
HDMB-3 p53-dn	3.9 μM	6 μM	dominant neg. form	ampl.

### Sensitivity to cell cycle inhibition is modulated by p53 status but not by MYCN levels

Interestingly, all cell lines harboring a p53 mutation, SK-N-FI, NLF and RH-41, displayed decreased sensitivity towards treatment with RO-3306 compared to p53 wt cells (data are summarized in Table 1). Neither down-regulation of MYCN in IMR5-TR-shNMYC cells (Supplementary Figure S3) nor induction of MYCN activity in SHEP MYCN-ER cells significantly altered cell viability (Supplementary Figure S4). Thus, the MYCN status did not affect sensitivity to RO-3306. The same p53-dependent effect was observed, when a pan cdk-inhibitor (JNJ-7706621) was used (Table 1 and Supplementary Figure S3–S5). Again, sensitivity to JNJ-7706621 did not depend on MYCN (Supplementary Figure S3C). Furthermore, when all cell lines were grouped according to their p53 status, cell lines without functional p53 displayed 1.7-fold higher resistance to RO-3306 and 1.8-fold higher resistance to JNJ-7706621 (Supplementary Table 1). Specificity of cdk1 inhibition by RO-3306 in NB cells was analyzed by interrogating the phosphorylation status of the cdk1 target protein phosphatase 1α (PP1α -Thr320) as well as phosphorylation of pRb at Ser 807/811, which is mainly governed by cdk4. In all cell lines except for p53-mutated SK-N-FI cells, a reduction of phosphorylated PP1α was observed after four hours following treatment with RO-3306, while RO-3306 did not affect pRb-phosphorylation (Supplementary Figure S6). Thus, NB cell viability can be significantly reduced by cdk-1 inhibition, while sensitivity to RO-3306 and JNJ-7706621 correlated with p53-mutational status rather than with MYCN levels.

### Sensitivity of NB cells to cdk1-inhibition is modulated by p53

**Figure 5:**
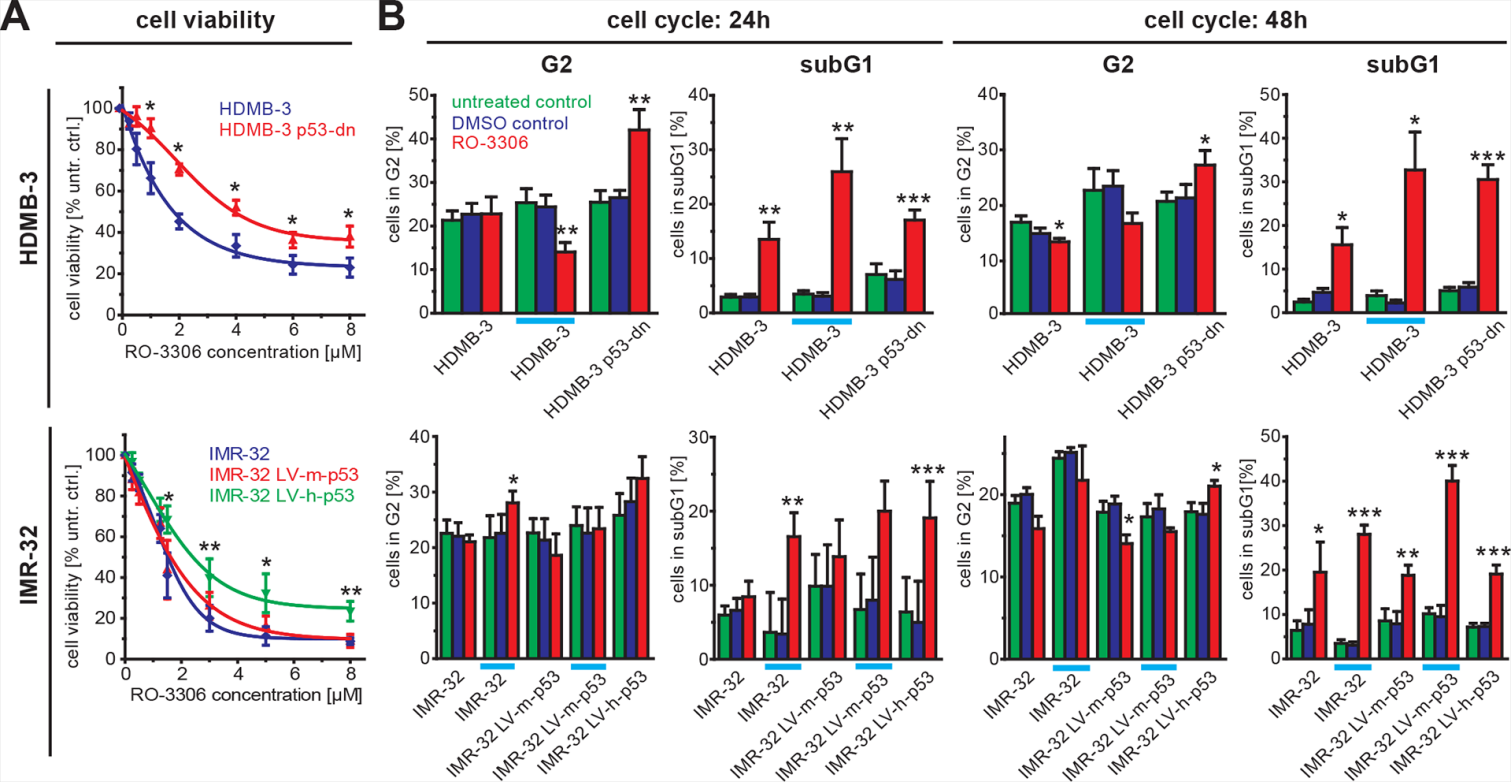
Sensitivity to RO-3306 is modulated by p53 functional status **A.** HDMB-3 medulloblastoma cells expressing a dominant negative p53 variant (HDMB-3 p53-dn) are more resistant to RO-3306 treatment than the parental HDMB-3 cells (upper part). Additionally, IMR32-NB cells harboring a shRNA against human p53 (IMR32-LV-h-p53) were less sensitive to RO-3306 compared to control cells transfected with shRNA directed against murine p53 (IMR32-LV-h-p53) and parental IMR-32 cells. **B.** Cell cycle analyses performed for the same cell panel as in (A) revealed a significant increase in G2 phase 48 hours after RO-3306 treatment only in the absence of functional p53. When control cells were treated at higher doses corresponding to the IC50 values of cells without functional p53 (indicated by an orange line), IMR-32 cells had also a significantly increased G2 fraction 48 hours after treatment with RO-3306. Again, in all cell lines RO-3306 caused an increase in the sub G1 fraction. Significant differences between untreated cells and inhibitor treated cells are depicted using the following code “***”: *p* < 0.001; “**”: *p* = 0.001−0.01; “*”: *p* = 0.01−0.05.

To further analyse the impact of the p53 mutational status on NB cell response to RO-3306, we made use of previously described isogenic IMR32 and NGP cells, in which p53 knock-down is mediated by shRNA [21]. These cells were designated IMR32-LV-h-p53 and NGP-LV-h-p53, respectively, and the results compared to parental cells or negative controls, which were stably transfected with shRNA against murine p53. Both cell lines also differ in the MDM2 status, as NGP cells harbor an amplification of MDM2, a negative regulator of p53. First, we excluded that knock-down of murine or human p53 had an effect on cdk1 or CCNB1 expression (Supplementary Figure S7). Interestingly, both p53 knock-down cell lines were more resistant to RO-3306 treatment when compared to the control cell lines and the parental cells. The fraction of viable IMR32-LV-h-p53 cells was also significantly higher at the highest concentration of RO-3306 tested (8 μM, Figure 5A), while NGP-LV-h-p53 were significantly more resistant than control cells at lower doses (Supplementary Figure S8A). To further delineate if p53 function was involved in RO-3306 mediated reduction of cell viability, we analyzed HD-MB3 medulloblastoma cells with or without expression of a dominant-negative form of p53 (HDMB3 p53-dn, [27]). Here, lack of functional p53 resulted in a twofold increased resistance to RO-3306 (IC50: 3.9 μM) compared to the parental cell line (IC50: 1.8 μM, Figure 5A). Taken together, cells with normal p53 status were more sensitive to RO-3306 treatment compared to their isogenic counterparts with either reduced levels or impaired function of p53.

### RO-3306 induces cell death and a p53 status dependent increase in G2 phase cells

As cdk1 is a cell cycle regulator promoting the transition from G2- to M-phase, we next investigated the effects of cdk1 inhibition on cell cycle distribution and apoptosis. Cell cycle analyses revealed an increase of the subG1-phase in our NB cell line panel 24 h and 48 h after RO-3306 treatment. This effect was significant in all cell lines independent of p53 mutational status (Figure 4B). Enhanced apoptosis could also be confirmed by an assay detecting isolated nucleosomes (Figure 4C). In p53-wt cells, activation of the p53-pathway by RO-3306 resulted in enhanced p21 expression after 24 hours, while p53-mutated cells failed to activate p21 (Figure 4D, Supplementary Figure S9). Additionally, the p53 downstream targets BAX and MDM2 were upregulated after cdk1 inhibition in p53 wt cells (Supplementary Figure S9). Instead, p53 mutated cell lines (SK-N-FI and NLF) presented with a significant increased fraction of cells in G2-phase. Similar results were also obtained in the model systems with reduced p53 levels (IMR32-LV-h-p53) and in cells expressing a dominant negative variant of p53 (HDMB3 p53-dn, Figure 5). Next, we assessed, if the observed G2 arrest induced by RO-3306 was dose-dependent. Treatment of parental IMR32 and HDMB3 cells with higher concentrations of RO-3306 did not increase the number of G2-phase cells after 48 hours (Figure 5B), while both the parental, MDM2-amplified, NGP cells as well as NGP-LV-h-p53 cells presented with a G2-phase arrest 24 h and 48 h (Supplementary Figure S8B). Thus, cell cycle analyses revealed that p53-deficiency modulates response to cdk1 inhibition by increasing the fraction of cells in G2, but that decreased p53-function does not impair RO-3306-induced apoptosis.

### RO-3306-induced apoptosis is caspase dependent

**Figure 6:**
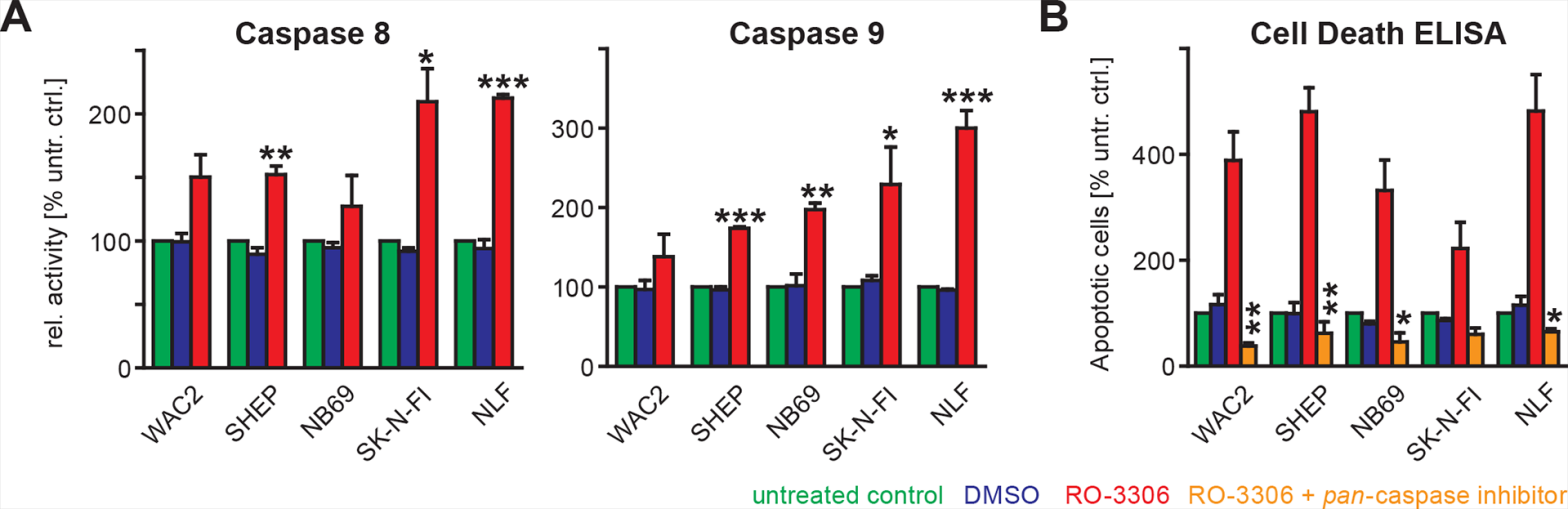
RO-3306 induced apoptosis is mediated by caspases **A.** Caspase-8 and -9 activities were investigated by colorimetric assays (“Caspase Glo”, Promega). RO-3306 caused elevated caspase-8 and -9 activities in all cell lines investigated after 48 hours and this increase was significant as indicated. **B.** RO-3306-induced apoptosis could be abrogated by co-treatment with a pan-caspase inhibitor, (Q-VD-OPh, used at a final concentration of 12.5 μM). This rescue effect was observed using a colorimetric assay (Cell Death ELISA, Roche) and confirmed by FACS-based cell cycle analyses (Supplementary Figure S10). Significances between RO-3306-treated cells and untreated cells (A) or between RO-3306 treated cells in the presence of absence of pan-caspase inhibitor are depicted using the following code: “***”: *p* < 0.001; “**”: *p* = 0.001−0.01; “*”: *p* = 0.01−0.05.

As treatment with RO-3306 resulted in an increased fraction of apoptotic cells independent of their p53 status, we next addressed involvement of the extrinsic and the intrinsic apoptotic pathway by monitoring caspase 8 and caspase 9 activity, respectively. Activation of both caspase 8 and caspase 9 upon treatment with RO-3306 was highest in the p53 mutated NB cell lines SK-N-FI and NLF (Figure 6A). This effect was confirmed by a rescue experiment, when co-administration of RO-3306 and a pan-caspase inhibitor, Q-VD-OPh, resulted in a significant reduction of the apoptotic response in all cell lines independent of the p53 status (Figure 6B, Supplementary Figure S10A). Only in NB69 cells, co-administration of RO-3306 and Q-VD-OPh resulted in a significant increase of cells in G2-phase in addition to suppression of apoptosis (Supplementary Figure S10B). Hence, canonical caspase-mediated apoptosis seems to be responsible for the observed cell death upon RO-3306 treatment.

## DISCUSSION

Uncontrolled proliferation plays a major role in cancer development and progression [28]. Under normal physiological conditions, cell cycle control is executed by a delicate interplay of cyclin dependent kinases (cdks) and their corresponding cyclins, whereas overexpression or amplification of cyclins and cdks is observed in various cancer types. Therefore, several cdk inhibitors were developed and entered early clinical phases, but those investigational drugs, including the pan cdk inhibitors flavopiridol and roscovitine, showed moderate effects and dose-limiting toxicities. Finding a therapeutic window was reported as a major difficulty [29]. In recent years, more specific inhibitors became available, which might have preferable anti-tumor characteristics compared to the broad spectrum inhibitors used before. Of note, a combined cdk4/cdk6 inhibitor, palbociclib, recently received “breakthrough therapy” status for the treatment of ER+/HER2- breast cancer. However, identification of the best suited inhibitor will depend on the tumor type and still is a challenge.

In neuroblastoma (NB), genomic amplifications of CCND1 [30] and cdk4 [31] were observed in primary tumors, albeit at low frequency. Nevertheless, cdk4 and CCND1 levels are found to be generally expressed at higher levels in NB than in other tumors [30]. Inhibition of cdk4 and cdk6 has also been shown promising activity in preclinical NB models [32]. Moreover, inactivation of cdk2 is synthetic lethal in MYCN-amplified tumor cells and cdk2 was also proposed as a potential target for NB therapy [7, 33]. Here, we confirm high expression of cdks as a feature of NB with unfavorable prognosis and additionally identify CCNB1/cdk1 expression as an MYCN-independent risk factor (Supplementary Figure S1). Both CCNB1 and cdk1 mRNAs are highly expressed in primary NB and this was correlated with reduced overall survival (Figure 1). NB cell lines derived from high-stage, aggressive tumors as well as the rhabdomyosarcoma cell line RH-41 all presented with high cdk1/CCNB1 expression independent of the MYCN amplification status (Figure 2). Induction of MYCN in a MYCN-normal cell line caused upregulation of cdk1/CCNB1 expression, while decreased MYCN expression in a MYCN-amplified cell line resulted in reduced cdk1/CCNB1 expression (Supplementary Figure S3B). However, the high expression levels of cdk1/CCNB1 in cell lines with normal MYCN (Figure 2), which are also found in primary tumors in the absence of MYCN amplification, left us to conclude that MYCN is a sufficient but not a necessary driver of cdk1/CCNB1 in neuroblastoma cells.

A prominent downstream target of the CCNB1/cdk1 complex is the tumor suppressor p53, which restricts uncontrolled cell growth by inducing cell cycle arrest, mainly in G1, or apoptosis. Inhibition of CCNB1/cdk1 reactivated and stabilized p53 in cells with functional loss of p53 [34]. Using sequence-specific siRNAs (Figure 3) or the quinolinyl thiazolinone derivative, RO-3306 ([14], Figure 4), to inhibit CCNB1/cdk1, we observed decreased cell viability and an increased number of apoptotic cells in all NB cell lines investigated. Interestingly, inhibitor efficiency depended on the p53 status, as cells with p53 mutation were more resistant to treatment with RO-3306. The impact of the p53 status on inhibitor efficiency could be confirmed in p53 knock down cells (Figure 5A). Additionally, higher inhibitor concentrations were required to decrease viability in a cell line (NGP) with functional inactivation of p53 due to MDM2 amplification. Increased resistance to cdk inhibition in the absence of functional p53 was not confined to neuroblastoma cells as shown in HDMB3 medulloblastoma cells expressing a dominant negative variant of p53 (Figure 5A). In all cell lines analyzed, p53 status did not affect CCNB1 or cdk1 expression mRNA level or protein levels. Thus, sensitivity to CCNB1 or cdk1 inhibition was modulated by functional p53, but was not dependent on the MYCN status.

The most prominent function of CCNB1 and cdk1 is to form a complex activating the serine/threonine kinase function of cdk1 thus regulating the transition from G2-phase to M-phase. As cdk1 is also sufficient to drive the entire cell cycle [35], we checked for specific changes in cell cycle distribution upon cdk1 inhibition. In cells with intact p53 signaling, apoptosis was the predominant response to cdk1 inhibition (Figure 4C). Our results add further evidence to the hypothesis that p53 status rather than MYCN expression governs the sensitivity to cdk inhibition (Supplementary Figure S3–S5) and, moreover, indicate that the cdk1/CCNB1 axis is important for the resistance to cdk inhibition by loss of functional p53 in neuroblastoma cells (Supplementary Table 1). Accumulation of p53 protein after cdk1 inhibition coincided with transcriptional upregulation of p53 targets, p21, BAX and MDM2 (Supplementary Figure S9). These results confirm earlier reports obtained upon RO-3306 treatment of AML cells [36]. It is also known that p53 can be phosphorylated by cdk1 at Ser315 and that this phosphorylation destabilizes p53 (ref. [34]). Therefore, earlier reports that cdk1 inhibition might function independently of the cellular p53 status, could possibly be attributed to unspecific inhibitors used [37]. In cells without functional p53 a significant increase in G2 phase was the major response to RO-3306 (Figure 4B, 5B), and this could be confirmed in cells expressing either a shRNA targeting p53 or a dominant negative form of p53. Mechanistically, this difference in the apoptotic response could not be attributed to differential activation of effector caspases, as caspase 8 and 9 were activated after 48 h. Caspase-9 phosphorylation by cdk1 has been described to protect cells from apoptosis during mitosis [38], while caspase-8 cleavage and thus activation is regulated by concerted action of cdk1 and polo-like kinase 1 (Plk1, ref. [39]). Consequently, a pan-caspase inhibitor could block activation of both caspase-8 and caspase-9 activation observed upon RO-3306 treatment and could significantly rescue cells from RO-3306-induced apoptosis independent of the p53 status (Figure 6). Thus, by a combination of loss-of-function and rescue experiments we could confirm p53 as well as caspase-8 and caspase-9 as important modulators of the response to cdk1-inhibition by RO-3306.

In summary, we here demonstrate that cdk1 inhibition by RO-3306 causes apoptosis in a panel of preclinical embryonal tumor models with wild-type 53 and that inhibitor efficiency was determined by p53 mutational status. Induction of apoptosis by cdk1-inhibition depended on functional p53 signaling, while in cells with p53 inactivation or in cells harboring TP53 mutations a significant G2 phase arrest was observed. Further investigation of these effects in *in vivo* models is warranted to evaluate the efficacy of targeting cdk1 as a novel tool in tumor therapy.

## SUPPLEMENTARY FIGURES AND TABLE



## Abbreviations

CCN: cyclin
cdks: cyclin-dependent kinases
MNA: MYCN-amplification
MYCN: v-myc myelocytomatosis viral related oncogene, neuroblastoma derived
NB: neuroblastoma
PP1α: protein phosphatase 1 alpha
Rb: retinoblastoma
wt: wild-type
