# Supplementary material for: Sensitivity to cdk1-inhibition is modulated by p53 status in preclinical models of embryonal tumors
Source: Oncotarget. 2015 May 11;6(17):15425–35. doi: 10.18632/oncotarget.3908 (PMC4558161; doi:10.18632/oncotarget.3908)
Supplement: Supplementary file 1 [file oncotarget-06-15425-s001.pdf]

SUPPLEMENTARY FIGURES AND TABLE

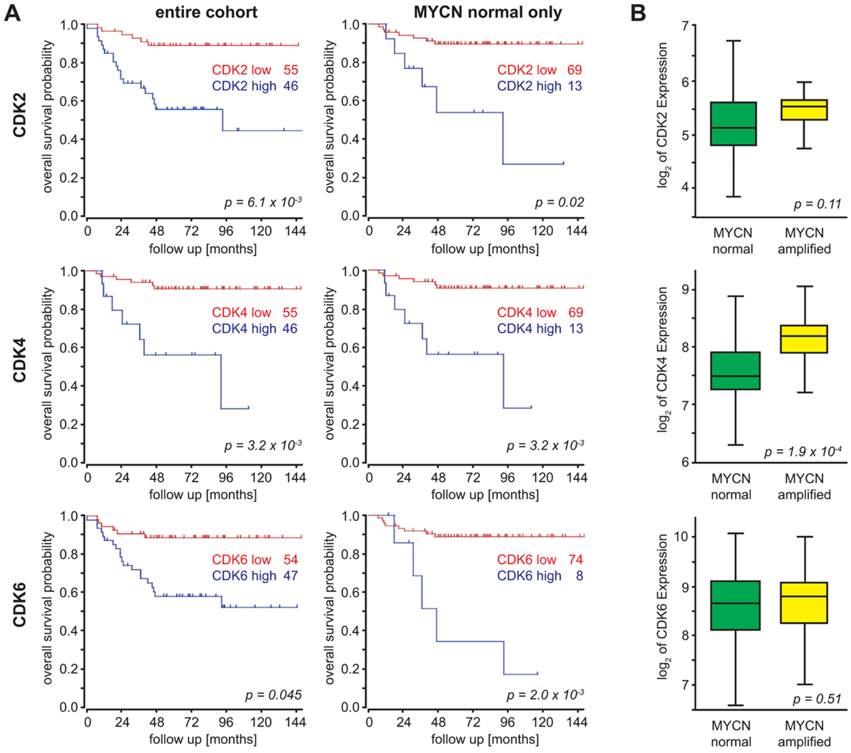

Supplementary Figure S1: cdk2, cdk4 and cdk6 expression are associated with overall survival and MYCN amplification. A. Kaplan Meier analyses of 101 NB revealed a correlation between overall survival and cdk2, cdk4 and cdk6 mRNA-expression, regardless of the MYCN status. The cohort was split into two groups with either "high" or "low" cdk expression and the numbers depict the respective group size, when the entire cohort (left) or the subset with normal MYCN status (right) were analyzed. *P*-values were adjusted for multiple testing of different group sizes using a Bonferroni correction implemented in the r2 visualisation module (r2.amc.nl). **B.** Box plots indicate an upregulation of cdk2, cdk4 and cdk6 in MYCN-amplified tumors, but this only reached statistical significance for cdk4.

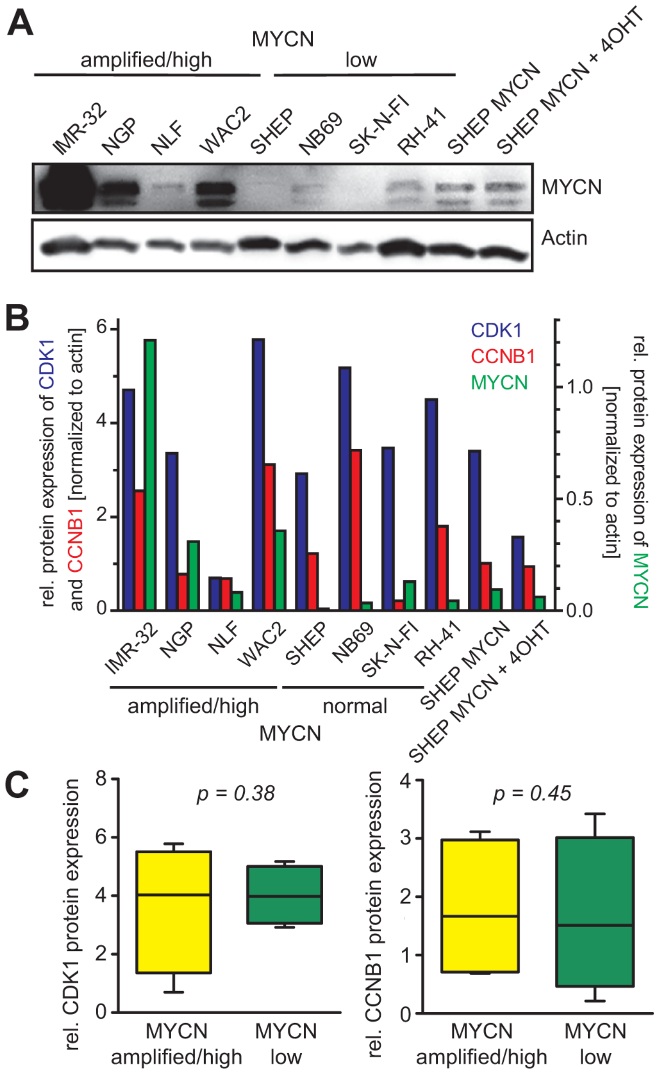

Supplementary Figure S2: cdk1 and CCNB1 expression are not significantly correlated with MYCN status in NB cell lines. A. MYCN protein expression levels in NB cell lines were detected by Western Blot analyses. **B, C.** Quantitative protein expression analyses showed no correlation between MYCN levels (green) and cdk1 (blue) or CCNB1 (red).

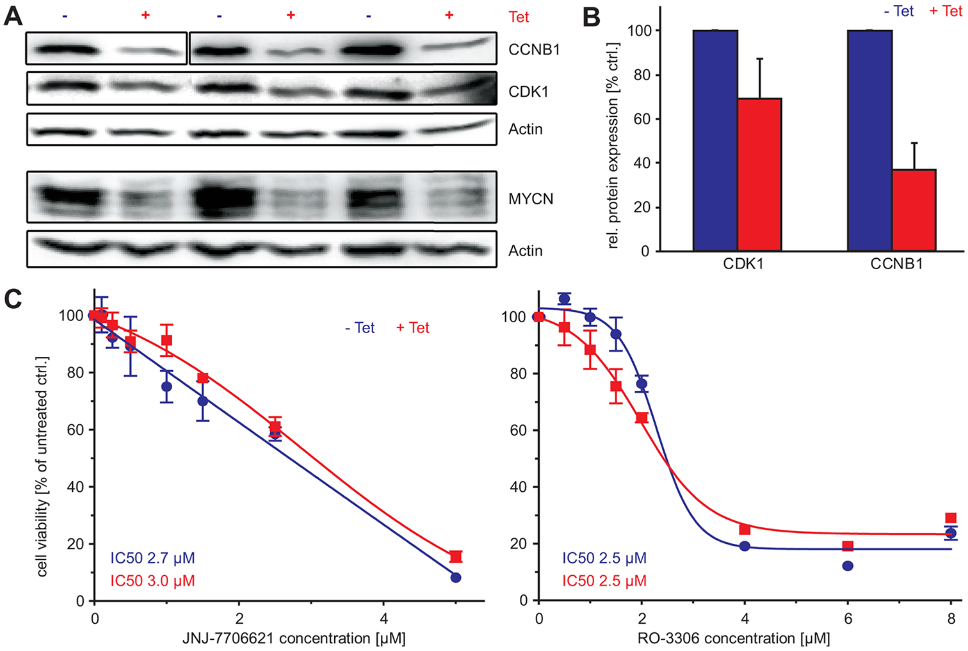

Supplementary Figure S3: Down-regulation of MYCN is associated with reduced expression of cdk1 and CCNB1 expression, but does not affect sensitivity to cdk inhibitors. A, B. Down-regulation of MYCN was achieved by addition of tetracycline (1 µg/ ml) to the culture media of IMR5-TR-shNMYC cells. Western Blot analysis was performed after treatment with tetracycline for 72 h. Down-regulation of MYCN is accompanied by reduced cdk1 and CCNB1 protein levels. **C.** Reduced MYCN levels (IMR5-TR-shNMYC, red curve) do not significantly alter sensitivity to the pan cdk inhibitor (JNJ-7706621, left) and to the specific cdk1 inhibitor (RO-3006, right). Cell viability was analyzed by MTT assays.

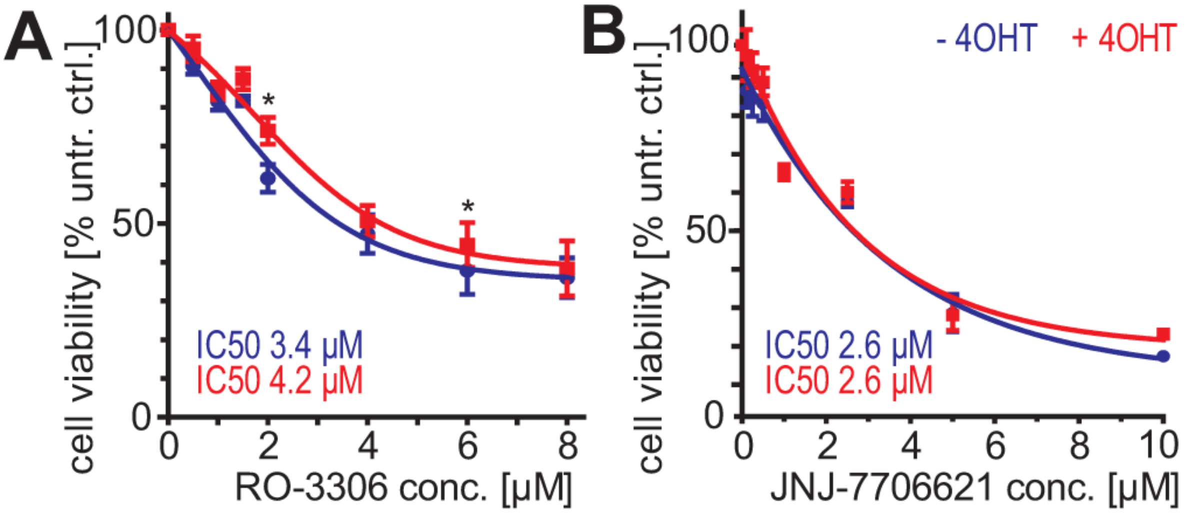

Supplementary Figure S4: Activation of MYCN in SHEP-MYCN cells does not significantly alter sensitivity to RO-3306. In SHEP-MYCN cells, MYCN activation can be achieved by addition of 4-OHT. Treatment of SHEP-MYCN cells with RO-3306 in the absence or presence of 4-OHT suggested a trend for decreased sensitivity when MYCN was activated, however, IC50 values were in a very narrow range (3.4 vs 4.2 µM). Cell viability was analyzed by MTT assays.

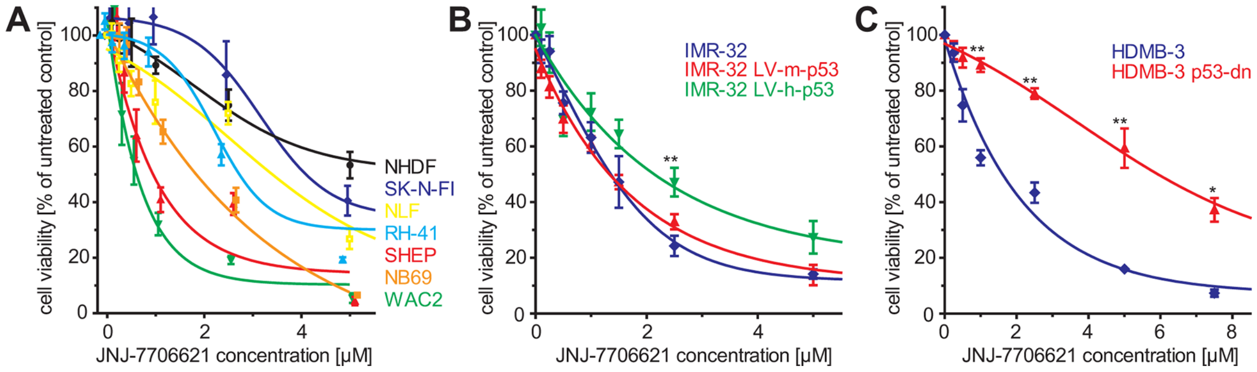

Supplementary Figure S5: The inhibitory effects of the pan cdk-inhibitor JNJ-7706621 on cell viability depends on the p53 status of the cells. A. JNJ-7706621 caused a reduction of cell viability after 48 h in all tested NB cell lines as well as in the rhabdomyosarcoma cell line (RH41) in a concentration dependent manner. Cell lines harboring mutated p53 (RH-41, SK-N-FI and NLF) were less susceptible to the inhibitor reflected by higher IC50 values (summarized in table 1). **B, C.** Down-regulation of p53 using an isogenic neuroblastoma cell lines model (IMR-LV-h-p53, B) or an isogenic medulloblastoma cell line model (HDMB3 p53 dn) resulted in reduced sensitivity to JNJ-7706621 compared to the respective parental cell lines harboring wt p53.

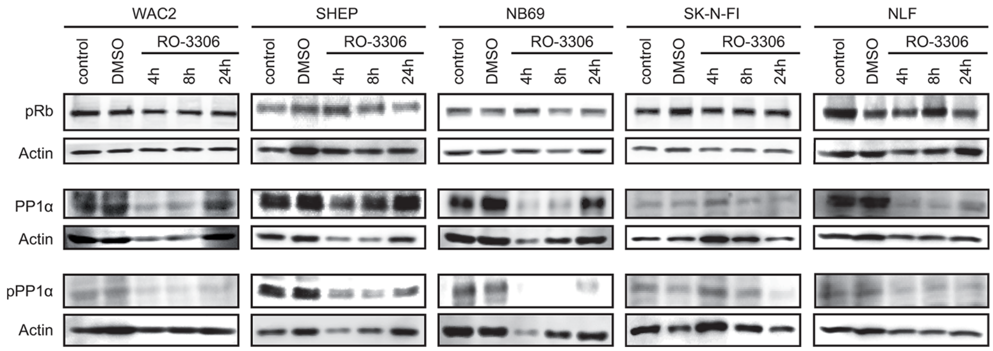

Supplementary Figure S6: RO-3306 specifically inhibits the kinase activity of cdk1, but not of cdk4. In five NB cell lines, the effects of RO-3306 on phosphorylation of the cdk-1 target protein, PP1α (PP1α-Thr320), and the cdk-4 specific phosphorylation sites of pRb (pRb-Ser 807/811) were monitored by Western Blot analyses over time as indicated. While total PP1α levels (designated as PP1a) were only transiently down-regulated after RO-3306 treatment in all cell lines investigated, phosphorylation of PP1α (designated as pPP1A) was still blocked after 24 hours in SK-N-FI and NLF cells. Reduction of PP1α phosphorylation was transiently blocked in WAC2, SHEP and NB69 cells. By contrast, we did not observe phosphorylation of Rb at cdk4-specific sites (indicated as pRb).

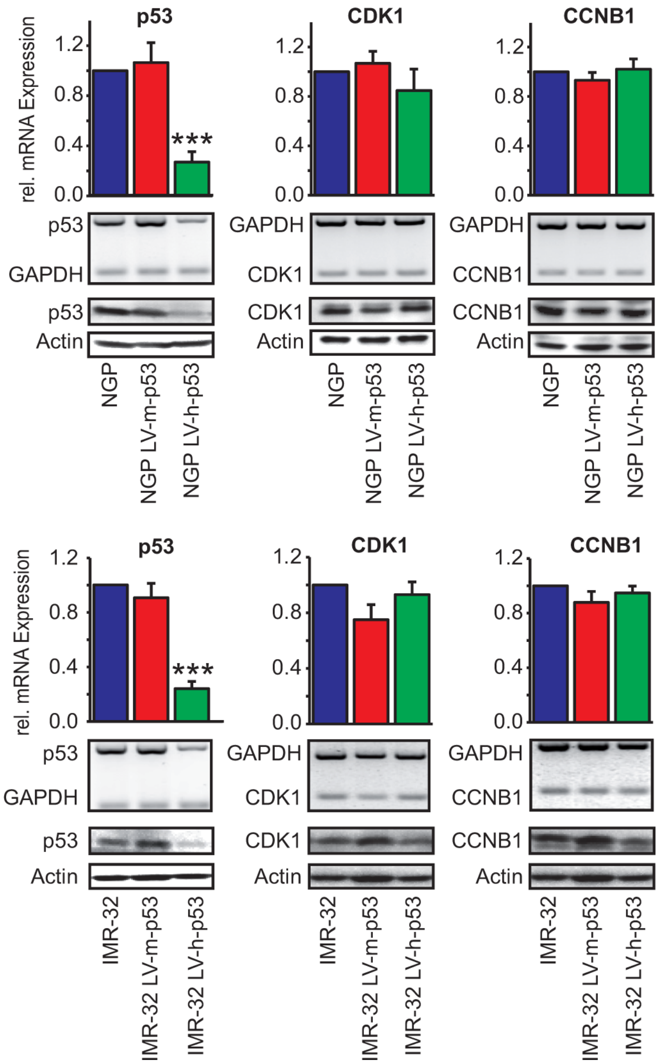

Supplementary Figure S7: Isogenic NB cells with variable p53 levels express comparable amounts of cdk1 and CCNB1. IMR-32 and NGP cells were previously engineered to stably express shRNA directed against human (LV-h-p53) or murine (LV-m-p53) p53 [21]. In these cell lines, p53, cdk1 and CCNB1 levels were investigated by RT-qPCR and Western Blot, respectively. While p53-levels were significantly decreased in the respective knock-down cells ($p < 0.001$), cdk1 and CCNB1 expression was unaltered.

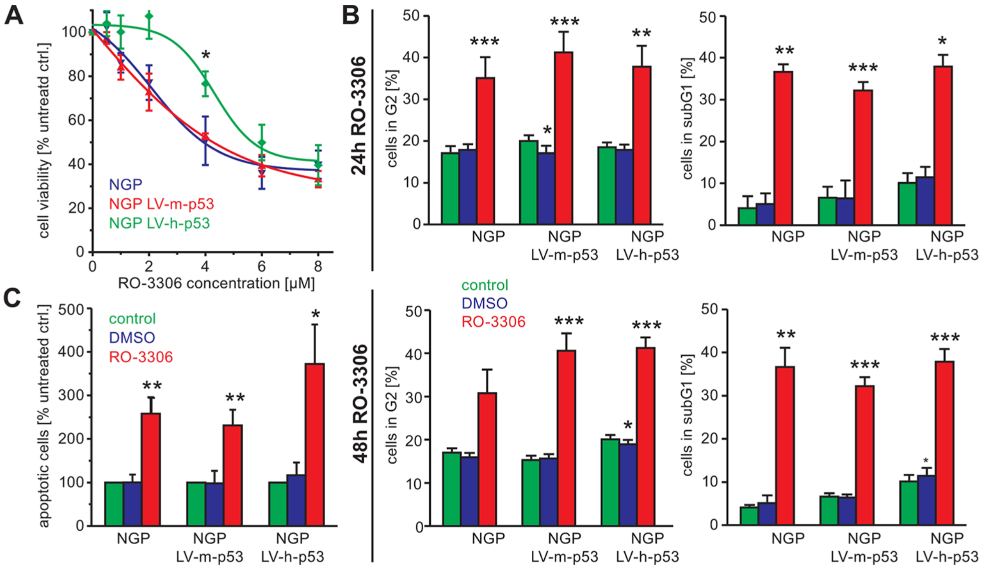

Supplementary Figure S8: In MDM2-amplified NGP cells, shRNA-mediated down-regulation of p53 modulated the anti-proliferative effects of RO-3306. A. Cell viability assays reveal that shRNA-mediated p53 knock-down in NGP cells (NGP-LV-h-p53) decreases the sensitivity to the cdk1 inhibitor, RO-3306, compared to control cells (NGP-LV-m-p53 and NGP). **B.** Regardless of the p53 status, NGP cells treated with RO-3306 for 24 or 48 hours present with an increase of G2-phase and of sub G1-phase cells. **C.** Significant induction of apoptosis in RO-3306 treated cells compared to untreated controls was validated by a colorimetric assay. Significances are indicated using the same code as in Figure 5.

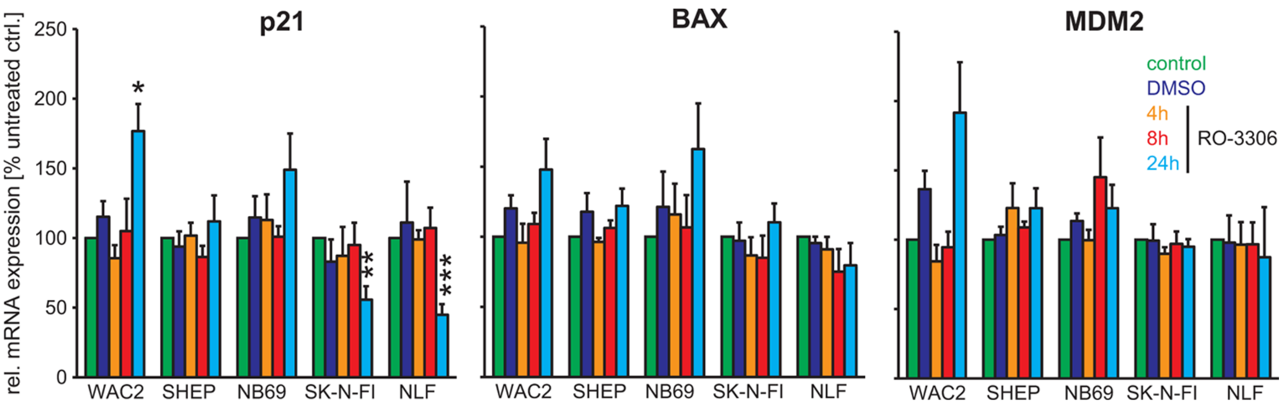

Supplementary Figure S9: Activation of p53 downstream targets by RO-3306. The mRNA profile of p53 downstream targets p21, BAX and MDM2 was investigated at indicated time points after treatment with RO-3306. While transcription of all three genes was upregulated by RO-3306 treatment, only p21 mRNA was significantly elevated at 24 hours in p53 wt WAC2 cells as revealed by quantification of semi-quantitative RT-PCR analyses (marked by an asterisk). Interestingly, p21 mRNA was significantly down-regulated in NB cells harboring a p53 mutation, SK-N-FI and NLF.

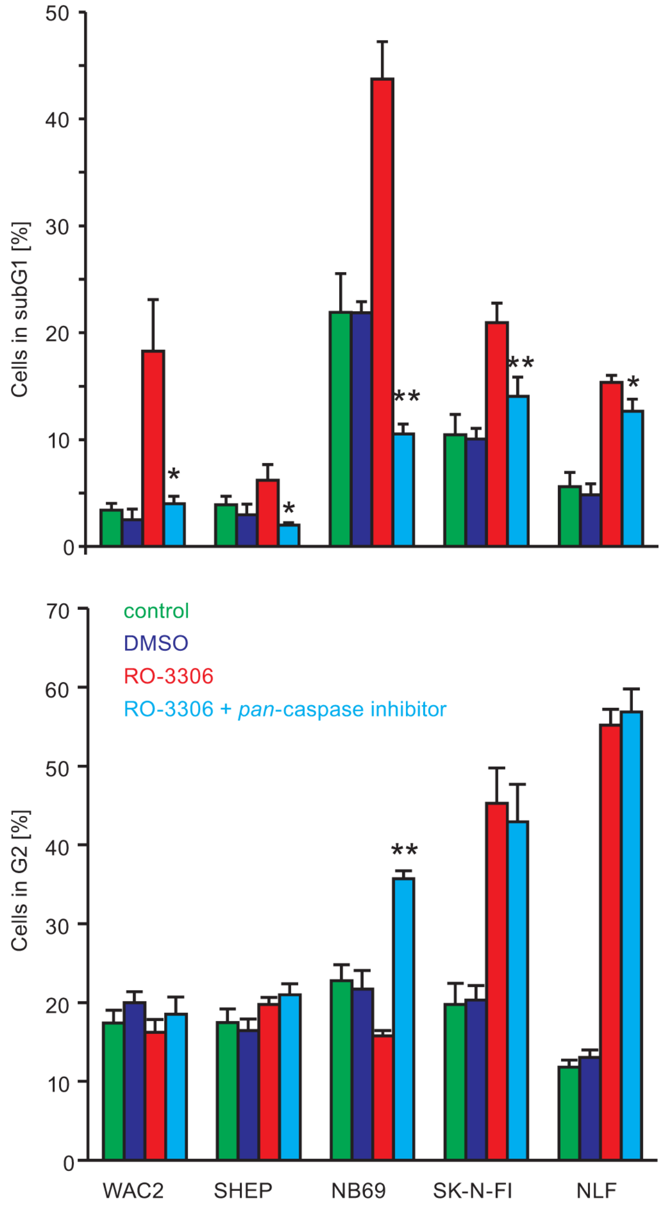

Supplementary Figure S10: Inhibition of caspases can prevent RO-3306 induced apoptosis. Cell cycle analyses revealed a significant reduction of subG1 phase in five NB cell lines, when cells simultaneously treated with RO-3306 and a pan Caspase inhibitor (Q-VD-OPh, used at a final concentration of 12.5 µM) were compared to cells treated with RO-3306 only. In NB69 cells, a significant increase of G2 phase cells was observed, when RO-3306 treated cells were additionally given Q-VD-OPh. Significance between RO-3306 treated cells in the presence of absence of pan-caspase inhibitor are depicted using the following code: “***”: $p < 0.001$; “**”: $p = 0.001–0.01$; “*”: $p = 0.01–0.05$.

Supplementary Table S1: Sensitivity of isogenic IMR32, NGP and HD-MB3 cells with normal and impaired p53 function to cdk1 inhibition by RO-3306 and to pan-cdk inhibition by JNJ-7706621. Numbers depict the IC50 values determined by MTT assay after treatment for 48 hours as indicated. "Rf"= resistance factor, which is the ratio of the IC50 value of cells with impaired p53 function (p53-) divided by the IC50 value of cells with normal p53 function (p53+).

	IMR32			NGP			HDMB3			all		
	IC50 [µM]	IC50 [µM]	Rf	IC50 [µM]	IC50 [µM]	Rf	IC50 [µM]	IC50 [µM]	Rf	IC50 [µM]	IC50 [µM]	Rf
**p53**	+	-		+	-						-	
**RO-3306**	*1.6*	*2.4*	*1.5*	*4.0*	*6.0*	*1.5*	*1.8*	*3.9*	*2.2*	*2.3*	*4.0*	*1.7*
**JNJ-7706621**	*1.3*	*2.1*	*1.6*	*3.1*	*3.1*	*1.0*	*1.6*	*6.0*	*3.7*	*1.8*	*3.3*	*1.8*
**$Rf_{RO-3306/}$ $Rf_{JNJ-7706621}$**			*0.9*			*1.5*			*0.6*			*0.9*
